# Mean Platelet Volume in Ocular Behçet's Disease

**DOI:** 10.1155/2013/215912

**Published:** 2013-10-22

**Authors:** Fatih Mehmet Türkcü, Abdullah Kürşat Cingü, Harun Yüksel, Yasin Çınar, Meltem Akkurt, Muhammed Şahin, Zeynep Özkurt, Alparslan Şahin, İhsan Çaça

**Affiliations:** ^1^Department of Ophthalmology, Faculty of Medicine, Dicle University, Diyarbakir, Turkey; ^2^Department of Dermatology, Faculty of Medicine, Dicle University, Diyarbakir, Turkey

## Abstract

*Objective*. To determine whether mean platelet volume (MPV) is an indicator of disease severity in ocular Behçet's Disease (BD). *Materials and Methods*. The study population was 30 newly diagnosed ocular BD patients who presented with active uveitis. These patients had no past history of smoking, drug use, or systemic diseases including diabetes mellitus, hypertension, cardiovascular disease, and renal disease. A control group consisting of 34 healthy individuals was included for comparison. MPV measurements were performed serially upon presentation with active uveitis and at one and three month thereafter in BD group whereas only at presentation in the controls. *Results*. Upon presentation with active uveitis, the mean MPV levels were 7.88 ± 1.14 femtoliters (fL) for BD group. During the posttreatment follow-up period at first and third months, BD patients demonstrated a mean MPV level of 7.71 ± 1.12 fL and 7.65 ± 1.04 fL, respectively. The mean MPV value of control group, was 8.39 ± 0.66 fL at presentation. Fluctuations in MPV values were not significant in the BD group, while there was a significant difference between the initial measurements of the BD and control groups. *Conclusion*. MPV measurement in ocular BD is not a predictive laboratory test to determine the clinical improvement in early stages following classical immunosuppressive treatment.

## 1. Introduction

Behçet's Disease (BD) is a multisystem inflammatory disease of unknown etiology with a relapsing and remitting clinical course. Among the criteria of International Study Group of BD uveitis is one of the major and common symptom particularly in certain regions [1–3]. BD patients may also have vascular involvement associated with thrombosis, yet the mechanism of thrombus formation has not clearly been elucidated [4, 5]. Endothelial cells play a significant role in homeostasis as they contribute to maintain vascular tone, the formation of clots, and induction of fibrinolysis. The tendency for thrombosis is not sufficiently explained by endothelial injury alone in BD patients. Thus coexistence of vasculitis and thrombosis is not evident in all BD patients [4–7]. 

Platelets have a central role in the pathogenesis of thrombo-occlusive diseases. Platelet function and activation is correlated with platelet volume [8, 9]. However, Mean Platelet Volume (MPV) is often disregarded by physicians even though it is included in the routine complete blood count (CBC) laboratory tests. In previous studies MPV values have been investigated in patients with BD, hypertension (HT), rheumatoid arthritis (RA), pseudoexfolation syndrome, retinal vein occlusion, diabetes mellitus (DM), and myocardial infarction (MI) [10–13]. Though there is no study about the MPV levels of ocular BD patients. In the current study we aimed to compare MPV values of healthy subjects and ocular BD patients during their active uveitis phase and at the time their disease activity suppressed with classical immunosuppressive treatment. 

## 2. Materials and Methods 

Thirty consecutive newly diagnosed active ocular BD patients and 34 healthy control subjects were recruited in the prospective, comparative study. Study group was diagnosed according to the guidelines established by the International Study Group. All patients were consulted with the rheumatology and dermatology departments [2]. Patients with any systemic or ocular thrombo-oclusive pathologies associated with BD were excluded from the study. Patients with glaucoma, DM, cerebrovascular disease, cardiovascular disease including MI, HT, hypercholesterolemia, malignancy, infectious diseases, liver or renal insufficiency, migraines, and Raynaud phenomenon, and patients with the history of hormone replacement therapy, antiplatelet therapy, antioxidant therapies, and immunosuppressive therapy were also excluded. This study was approved by the local ethics committee, and all subjects provided informed consent before they participated in the trial.

All participants received a complete ocular examination including best-corrected visual acuity on a Snellen scale, slit-lamp biomicroscopy, intraocular pressures (IOP) measurement with Goldmann applanation tonometry, and indirect ophthalmoscopy. 

All BD patients had panuveitis without retinal vascular occlusion. Active uveitis were defined as having cells in anterior chamber or vitreous, macular edema higher than 250 *μ*m and retinal vasculitis. Systemic corticosteroids together with conventional immunomodulatory agents (1–2.5 mg/kg per day azathioprine and/or 3–5 mg/kg per day cyclosporine A) were given to the patients according to their severity of uveitis. Those cases with sustained or increased disease activity during follow-up period were excluded from the study. The remaining patients were followed up for at least 3 months.

### 2.1. MPV Assessment

Complete Blood Count (CBC) analyses were performed with the Beckman Coulter Gen-S automated analyzer (High Wycombe, UK). In accordance with hospital laboratory policy, the CBC was performed within one hour of sample collection. We measured MPV in a blood sample collected in citrate in order to avoid the platelet swelling induced by EDTA.

### 2.2. Statistical Analysis

Statistical Package for Social Sciences (SPSS) version 15.0 was used to execute data analyses. One-sample Kolmogorov Smirnov test was performed to view the data distribution. Because the data were distributed normally, repeated measures of ANOVA test were used for variance analyses. Differences between numeric variables were tested via Student's *t*-test. All data were described as mean ± standard deviation. *P* values less than 0.05 were considered statistically significant. 

## 3. Results

The mean age of the BD patients and control subjects were 31.2 ± 7.9 years and 29.9 ± 7.9 years, respectively. The male to female ratio was 16 : 14 in the BD group and 15 : 19 in the control group. Upon comparing the study and control groups, there were no statistically significant differences in age (*P* = 0.484) and sex (*P* = 0.314). The initial mean MPV value in BD group was significantly lower than that of the control group (*P* = 0.037). There were no significant differences in the mean MPV values of BDs group in the first and third month levels when compared with the active uveitis phase (Table 1). 

## 4. Discussion

The mechanism causing thrombosis in BD has not yet been elucidated. Even though BD patients demonstrate endothelial dysfunction and abnormal fibrinolysis, a specific molecular defect that is involved in thrombosis has not yet been discovered [6, 14, 15]. Gene mutations in various haemostatic proteins such as protein C, protein S and factor *V* have been studied to understand the pathogenesis of thrombosis. It was determined that these factors, when mutated, may result in vasculitic dysfunction [15, 16]. Impairment in endothelial function is also observed in diseases characterized by chronic vasculitis and active inflammation such as BD. The major histopathological feature described in BD disease is vasculitis with prominent neutrophil and monocyte infiltration in the perivascular region with or without vessel wall fibrin deposition [17, 18]. Although the exact pathogenic mechanism regarding the formation of vascular lesions in BD remains unclear, endothelial dysfunction is thought to play an important role in the development of thrombosis. Besides, histological evidence of vasculitis with endothelial cell activation or injury is a distinguishing feature of BD [19]. 

Platelets have a central role in the pathogenesis thrombosis. So laboratory tests to analyze platelet function give some indirect information about the thrombotic events. One way to quantify platelet dysfunction is the evaluation of MPV value, which measures platelet size and activity. Correlations between increased MPV values and some thrombotic diseases such as deep vein thrombosis, acute MI, or acute ischemic cerebrovascular events have previously been reported [8, 11]. These clinical consequences of high MPV were explained by the fact that larger platelets are more reactive and more prone to aggregate, and consequently results in endothelial dysfunction [8, 17, 20, 21]. Specifically, larger platelets store and release higher amounts of serotonin, *β*-thromboglobulin, and thromboxane A_2_ [21, 22]. We can analyze endothelial dysfunction via some direct tests like flow-mediated dilation (FMD). However, this method is so expensive and technically challenging. Alternatively, as an indirect way to show endothelial dysfunction is measuring the MPV which is cost-effective and automated routine laboratory measurement. 

In our study, we found that MPV values were significantly lower in patients with BD in comparison with the control group. Yet, there were no differences in the active uveitis phase MPV levels of the BD group who were started with classical immunosuppressive treatment with the one and third month follow-up MPV measurements. These results suggest that there is a continuous vascular assault that sustains vasculitic lesions in BD as MPV values remain unchanged during the early treatment period in which disease activity was decreased under classical immunosuppressive therapy. However, increased platelet reactivity alone could not be blamed in the formation of vascular lesions in BD as its pathogenesis is more complex and multifactorial. 

Similar to our results Lee and Kim reported lower MPV values in their BD patients when compared with their control group [23]. Distinctly their study population also includes BD patients with thrombosis and they did not find any significant difference between the subgroups in MPV levels.

In contrast to our study Acikgoz and Karincaoglu reported increased MPV levels in BD patients when compared with health controls [7]. However, in their study while there was significantly higher levels of MPV in thrombotic BD patients they did not find any difference between active and inactive BD patients in MPV. Another study performed by Ricart et al. did not reveal any effect of the presence of posterior uveitis or thrombosis on MPV values in BD [24]. Yet it is important to note that the posterior uveitis subgroup in their study were not in the active phase and most of them had already received immunosuppressant therapy. Moreover, these patients had an average of 4.2 ± 3.1 years between the time of uveitis attack and the time that their MPV levels were evaluated [24]. 

Unlike the previous literature, in the current study we included only ocular BD patients with active panuveitis attack without initial medications that might impair MPV measurements. In a previous study improvement in endothelial function in active BD patients was demonstrated with FMD following systemic corticosteroid therapy [25]. In the current study we investigated the effect of classical immunosuppressive therapy on endothelial function in ocular BD patients with active panuveitis by measuring MPV levels. Besides in other inflammatory processes like Rheumatoid Arthritis and Ankylosing Spondylitis MPV levels were found to be lower than controls and significantly increased after systemic immunosuppressive treatment [13]. These data suggested that MPV values might decrease with steroid or immunosuppressive treatment in inflammatory diseases. However, in the current study we did not find any significant change in MPV value in the early treatment period with classical immunosuppressive therapy. Upon this statement one may conclude that MPV values would also be affected by some other variables besides immunosuppressive treatment. 

Our study demonstrated that patients with active ocular BD have lower MPV values then in healthy control subjects. To our knowledge this is the first study that concludes that there is no change in MPV values in BD patients during the active uveitis phase and 3 months thereafter with decreased disease activity under immunosuppressive treatment. 

There are some limitations that need to be mentioned regarding the current study. First we cannot give any information about the differences in MPV values of ocular and nonocular BD patients because of the study design. Second we could only give the early changes in MPV following classical immunosuppressive treatment in our study group. Nevertheless, our results suggest that MPV measurement in ocular BD is not a predictive laboratory test to determine the clinical improvement documented by ophthalmological examination following immunosuppressive treatment. Further studies are needed to determine long term changes in MPV levels following immunosuppressive treatment in ocular BD patients.

## Figures and Tables

**Table 1:** Mean platelet volume (MPV) in groups.

	Control	Active uveitis	Month 1	Month 3
MPV (fL)	8.39 ± 0.66^a,b,c^	7.88 ± 1.14^f^	7.71 ± 1.12^d^	7.65 ± 1.04^e^

^a^Compared with active uveitis and control group (*P* = 0.037), ^b^compared with first month MPV value and control group (*P* = 0.006), ^c^compared with third month MPV value and control group (*P* = 0.002), ^d^compared with first month MPV value and active uveitis (*P* = 0.210), ^e^compared with third month MPV value and active uveitis (*P* = 0.117), ^f^compared with first and third month MPV value (*P* = 0.666).
